# Shank3 related muscular hypotonia is accompanied by increased intracellular calcium concentrations and ion channel dysregulation in striated muscle tissue

**DOI:** 10.3389/fcell.2023.1243299

**Published:** 2023-09-06

**Authors:** Berra Yildiz, Lisa Schiedt, Medhanie Mulaw, Jürgen Bockmann, Sarah Jesse, Anne-Kathrin Lutz, Tobias M. Boeckers

**Affiliations:** ^1^ Institute for Anatomy and Cell Biology, Ulm University, Ulm, Germany; ^2^ International Graduate School in Molecular Medicine, IGradU, Ulm, Germany; ^3^ Unit for Single-cell Genomics, Medical Faculty, Ulm University, Ulm, Germany; ^4^ Neurologie, Universitätsklinikum Ulm, Ulm, Germany; ^5^ Deutsches Zentrum für Neurodegenerative Erkrankungen, Ulm Site, Ulm, Germany

**Keywords:** ASD, Shank3, muscular hypotonia, neurodevelopmental disorders, RNA-sequencing, immunohistochemistry, western blot

## Abstract

Phelan-McDermid syndrome (PMS) is a syndromic form of Autism Spectrum Disorders (ASD) classified as a rare genetic neurodevelopmental disorder featuring global developmental delay, absent or delayed speech, ASD-like behaviour and neonatal skeletal muscle hypotonia. PMS is caused by a heterozygous deletion of the distal end of chromosome 22q13.3 or *SHANK3* mutations. We analyzed striated muscles of newborn *Shank3Δ11(−/−)* animals and found a significant enlargement of the sarcoplasmic reticulum as previously seen in adult *Shank3Δ11(−/−)* mice, indicative of a Shank3-dependent and not compensatory mechanism for this structural alteration. We analyzed transcriptional differences by RNA-sequencing of muscle tissue of neonatal *Shank3Δ11(−/−)* mice and compared those to *Shank3(+/+)* controls. We found significant differences in gene expression of ion channels crucial for muscle contraction and for molecules involved in calcium ion regulation. In addition, calcium storage- [i.e., Calsequestrin (CSQ)], calcium secretion- and calcium-related signaling-proteins were found to be affected. By immunostainings and Western blot analyses we could confirm these findings both in *Shank3Δ11(−/−)* mice and PMS patient muscle tissue. Moreover, alterations could be induced *in vitro* by the selective downregulation of Shank3 in C2C12 myotubes. Our results emphasize that SHANK3 levels directly or indirectly regulate calcium homeostasis in a cell autonomous manner that might contribute to muscular hypotonia especially seen in the newborn.

## 1 Introduction

Autism Spectrum Disorders (ASD) encompass a vast range of neuropsychiatric diseases characterized by social and communication impairments, as well as repetitive and restricted behaviors, which can vary in severity among individuals (Grzadzinski et al., 2013). Worldwide, nearly one in every 100 children is diagnosed with ASD, which can be detected as early as at 18–24 months of age (Zeidan et al., 2022). The etiology of ASD is still not known, however genetic (Geschwind, 2008; Chaste and Leboyer, 2012), epigenetic (Loke et al., 2015) and environmental factors (Grabrucker, 2013) contribute to the emergence of the symptoms. More than 100 genes are related to ASD (Leblond et al., 2014). Among them, 1% of all ASD instances are caused by mutations in the scaffolding proteins Src homology 3 domain and multiple ankyrin repeat domain (SHANK)-genes, also known as proline-rich synapse associated proteins (ProSAP), including Shank1/ProSAP3, Shank2/ProSAP1 and Shank3/ProSAP2 (Boeckers et al., 1999; Naisbitt et al., 1999; Leblond et al., 2014). Within the SHANK protein family, a mutation in *SHANK3* reveals the most severe phenotype, which affects about 2% of individuals with ASD and intellectual disability (ID) (Leblond et al., 2014). *SHANK3* is located on chromosome 22q13.3, whose mutation leads to a haploinsufficiency of the SHANK3 protein and causes a rare genetic developmental disorder, called Phelan-McDermid syndrome (Durand et al., 2007; Delahaye et al., 2009; Sarasua et al., 2011; Phelan and McDermid, 2012). PMS is a syndromic form of ASD (Phelan and McDermid, 2012) which is clinically characterized by global developmental delay, absent or delayed speech, ASD-like behavior, and neonatal skeletal muscle hypotonia (Bonaglia et al., 2006). The hypotonia is of special importance since it appears directly after birth. Interestingly, Shank3 mutant macaques demonstrate reduced pulling force, while Shank3 KO mice exhibit impaired wire-hanging time, endurance, and motor coordination, analogous to the hypotonia observed in PMS patients (Zhou et al., 2019; Lutz et al., 2020; Bauer et al., 2023). We recently discovered SHANK3 expression in human and mouse sarcomeres and in neuromuscular junctions (NMJ) (Lutz et al., 2020). In muscle cells SHANK3 and α-Actinin form a complex localized at Z-discs. We further found increased calsequestrin (CSQ) levels in the sarcoplasmic reticulum (SR), which correlates with an enlargement of the SR in ultrastructural analysis in PMS patients and adult *Shank3Δ11(−/−)* mice (Lutz et al., 2020).

We interpreted our SR findings as a compensatory mechanism to eventually provide more calcium ions for a better neuromuscular coupling in Shank3 deficient striated muscle cells. To test this hypothesis, we were interested in transcriptional, biochemical and ultrastructural alterations of striated muscle tissue of neonatal P1 mice before final maturation and usage. To this end, we performed electron microscopy, calcium measurements and transcriptome analysis to examine differently regulated genes in *Shank3Δ11(−/−)* mice muscle (compared to *Shank3(+/+)* mice) and corroborated the RNA sequencing results at the protein level. Interestingly, we found an upregulation of intracellular calcium levels and enlarged CSQ positive SR structures already at this developmental stage. Moreover, we could identify a direct or indirect role of SHANK3 levels on transmembrane receptor channel expression as well as on calcium storage-, release- or calcium-related signaling-proteins. Our data indicate that early muscular hypotonia is due to alterations of Shank3 dependent protein expression that occurs at very early developmental time points. This may contribute to the emergence of early muscle hypotonia in neonatal PMS patients.

## 2 Materials and methods

### 2.1 Ethical statement

The review board of the Land Baden-Württemberg got ethical permission for the animal studies, Permit Numbers o.103-12 and z.103 TschB:W. The studies were carried out in accordance with rules established by the Federal Government of Germany, the National Institutes of Health, and the Max Planck Society for the welfare of experimental animals. Prior to sampling, all human donors or their guardians provided informed consent. The Ulm University Ethics Committee granted approval for this study, which was carried out in conformity with institutional and national guidelines and regulations (proposal numbers 208/16 and 265/12).

### 2.2 Animal housing

The animal research center at Ulm University provided wildtype C57BL/6 mice (*Mus musculus*). Following heterozygous C57BL/6JRj mating, knock-out Pro2 KO GVO *Shank3Δ11(−/−)* mice were obtained as explicated by (Schmeisser et al., 2012). The homozygous mice that were obtained have been described regarding Shank3 isoform expression in (Lutz et al., 2020 and Lutz et al., 2022). The animals were kept in a pathogen-free environment under standard laboratory conditions with constant access to food and water, an average temperature of 22°C, and a 12 h/12 h light/dark cycle. For biochemical analyses post-natal day 0–1 (P0-1) mice were genotyped and divided into two groups: wildtype *Shank3(+/+)* or knock-out *Shank3Δ11(−/−)* mice.

### 2.3 Electron microscopy

The sarcoplasmic reticulum analysis was performed on muscular tissue from three P0-1 *Shank3Δ11(+/+)* and *Shank3Δ11(−/−)* mice. Gastrocnemius muscles were dissected, washed with DPBS (Gibco) and fixed with pre-cooled 2% paraformaldehyde (PFA, pH 7.3, Merck), 2.5% Glutaraldehyde (GA, Plano Agar), 1% sucrose (Merck) in 0.1 M cacodylate (Agar Scientific) at 4°C for 24 h. The following embedding and cutting procedures were kindly performed by the Department of Electron microscopy of Ulm University. After being post-fixed in 2% aqueous osmium tetroxide (Fluka), the muscle tissues were washed three times with DPBS and treated with 0.1 M sodium cacodylate. The samples were then embedded in an epoxy embedding medium (Fluka). To determine the region of interest, semi-thin sections were cut and stained with toluidine blue staining solution (1% toluidine blue (Fluka), 1% Na-borate (Sigma) in water). The resin blocks were trimmed, cut into 80 nm ultra-thin sections, and placed on 300 mesh copper grids (Plano). 0.3% lead citrate was used to contrast the sections (Plano Agar). A Veleta camera and a JEOL 1400 Transmission Electron Microscope (TEM) were used to capture images (Olympus).

### 2.4 BAPTA measurement

The calcium measurement analysis was performed in accordance with the previously published protocol (Lamboley et al., 2015). Briefly, the gastrocnemius muscle from five P0-1 *Shank3(+/+)* and *Shank3Δ11(−/−)* mice was dissected and equilibrated in Ringers solution (146 mM NaCl, 5 mM KCl, 2 mM CaCl2, 1 mM MgCl2, 10 mM HEPES, pH 7.4) at RT for 10 min. The muscles were dried, weighted, and mechanically homogenized in measurement solution (120 mM KCl, 2 mM Hepes pH 8.0, 0.15 mM BAPTA) utilizing an electric tissue grinder. Then 0.5% sodium dodecyl sulfate (SDS, Roth) was added and centrifuged at 14,000 rpm at 6°C for 45 min. A microplate reader was used to measure the BAPTA and EDTA absorbance in the supernatant at 310 nm, which indicates the total calcium concentration in the skeletal muscle.

### 2.5 Immunohistochemistry

For immunostaining of the muscle, gastrocnemius muscles of P0-1 mice were dissected, lodged in O.C.T. (Tissue-Tek) and incubated in ice-cold 2-Methylbutan (Roth) for 5 min and in liquid nitrogen for 10 min. The muscles were cut into 7 µm thick longitudinal sections using a Leica CM1950 cryostat and collected on microscopy slides. Tissues were fixed with cold acetone (Sigma-Aldrich) for 10 min at −20°C. After 3 times rinsing with DPBS+/+ (Gibco) each time for 5 min, the sections were treated either by antigen retrieval with pre-warm 0.1 M citrate bufer (pH 6) at room temperature (RT) for 20 min (for anti-Cav1.3 (ms, 1:500, Abcam)) or were straightly blocked with 10% goat-serum (Millipore), 5% FBS (Gibco) and 0.2% Triton X-100 (Roche) diluted in 1x DPBS at RT for 4 h. Sections were then immunolabeled with the following primary antibodies (anti-SHANK3 (rb, 1:500, homemade, (Schmeisser, 2012)), anti-α-Actinin2 (ms, 1:500, Sigma A7811), anti-α-Actinin2 (rb, 1:500, Invitrogen PA5-27863), anti-Calsequestrin (ms, 1:500, Thermo Fisher MA3-913), anti-RyR (rb, 1:500, Abcam ab219798), anti-DHPR (ms, 1:500, Abcam ab2862), anti-PTK6 (ms, 1:500, Santa cruz sc-166171), anti-Cav1.3 (ms, 1:500, Abcam ab85491), anti-KCKN18 (rb, 1:500, ThermoFisher PA5-114308)) in blocking buffer and kept for 48 h at 4°C. The tissues were gently washed with DPBS+/+ 3 times each for 5 min. Secondary antibodies (Alexa Fluor 488 donkey anti-rabbit, Alexa Fluor 594 donkey anti-rabbit, Alexa Fluor 488 donkey anti-mouse, Alexa Fluor 594 donkey anti-mouse) were obtained from Jackson ImmunoResearch Laboratories, diluted 1:1000 in DPBS+/+ (Gibco) and incubated light protected for 2 h at RT. Following the same washing steps, tissue sections were mounted with ProLong Gold Antifade reagent with DAPI (Invitrogen). Images were acquired with a resolution of 1024 × 1024 pixels using the Leica TCS SPE II confocal microscope (Wetzlar, Germany).

Our control group consists of patients with mitochondriopathy (CNTL child (age of biopsy: 3 years)) or chronic pain syndrome (CNTL young (age of biopsy: 22 years), CNTL1 adult (age of biopsy: 40 years), CNTL2 adult (age of biopsy: 32 years)), who underwent a muscular biopsy, however, they revealed neither myopathic abnormalities nor mutations.

PMS child (age of biopsy: 2 years) had a mutation on chromosome 22 of 3.27 Mb, including the Shank3 gene. This child was diagnosed with PMS and showed global developmental delay, autistic features, microcephaly, ataxic gait, and impaired bimanual coordination (Status as at 2020). As of 2023, the PMS child continues to exhibit muscular hypotonia.

PMS young (age of biopsy: 20 years) harbored a deletion on chromosomal region 22q13.3 and displayed PMS along with mental retardation (Status as at 2020). As of 2023, PMS young has shown a general regress in clinical manifestations.

PMS adult (age of biopsy: 58 years) exhibited a translocation between chromosome 1 and chromosome 22, specifically at the breakpoint within the Shank3 gene and was diagnosed with PMS, displayed delays in development and speech (Status as at 2020). As of 2023, muscular hypotonia is no longer present in PMS adult.

Cryosections of human muscle biopsy were obtained from vastus lateralis (CNTL child, CNTL young, CNTL1 adult, CNTL2 adult, PMS child, PMS young and PMS adult), tibialis anterioris (CNTL young) and brachialis (CNTL2 adult). The subsequent staining was carried out as described above.

### 2.6 Transfection, differentiation and treatment of C2C12 mouse myoblast cells

To passage commercial C2C12 Mouse Myoblasts at 80% confluency, TrypLE (Gibco) was incubated for 2 min at 37°C. After gently knocking the flask, 5 mL of 1× DPBS (Gibco) was added. Cells were then collected in a falcon, centrifuged at 300 g for 2 min, and resuspended in DMEM + Glutamax (Gibco) with 20% fetal bovine serum (FBS) (Gibco) and 1% antibiotic-antimycotic (Thermo Fisher Scientific). 22,000 cells were plated in Growth-Factor-Reduced-Matrigel-coated (Corning) 35-µm dish (Ibidi). The day after, the myoblasts were transfected using Fugene HD Transfection Reagent (Fugene) with a plasmid containing GFP only (pSUPER-GFP) serving as control, a plasmid containing a siRNA for SHANK3 and GFP (Verpelli et al., 2011) (labeled as “Shank3 KD (knock down)” or pCMV R-CEPIA1er (Plasmid #58216, Addgene) and were kept in DMEM + Glutamax (Gibco) for 6 h. Afterwards, the medium was switched back to DMEM + Glutamax (Gibco) with 20% fetal bovine serum (FBS) (Gibco) and 1% antibiotic-antimycotic (Thermo Fisher Scientific). At 100% confluency medium was changed to Differentiation Medium [DMEM + Glutamax (Gibco), 2% Horse-Serum (Thermo Fisher Scientific) and 1% antibiotic-antimycotic (Thermo Fisher Scientific)]. After being differentiated for 14 days, the cells were either fixed with 4% PFA (Merck) or treated with 200 µM 4-Chlor-3-methylphenol (4CmC, Sigma) dissolved in water for 48 h.

### 2.7 Western blotting

The gastrocnemius muscle of P0-1 mice were mechanically homogenized in modified RIPA buffer (10 mM Tris–HCl pH 7.4 (AppliChem), 0.1% sodium dodecyl sulfate (SDS, Roth), 1% Triton X-100 (Roche), 1% sodiumdeoxycholate (Merck), 5 mM EDTA (Sigma), PhosphoSTOP™ (Roche) and Complete™ Mini EDTA-free Protease Inhibitor Cocktail (Roche)) using an electric tissue grinder before being lysed on ice for 15 min and followed by a sonification step of 10 pulses for 10s with 63% power. After incubation on ice for 30 min the lysates were centrifuged at 13,000 rpm at 4 °C for 10 min. The protein concentration was determined using a Bradford assay. Duplicates of 20 µL of 150 mM NaCl (Merck), 2 µL of the vortexed sample and 200 µL Bradford solution ((ethanol (95%, Roth), phosphoric acid (85%, VWR), Serva Blue (Serva) solved in distilled water) were placed in 96-well plates (Sarstedt), and measured at 595 nm absorbance using a microplate reader (CytationTM 3 Cell Imaging Multi-Mode Reader). After determining the protein concentration of the samples, same amount of protein was pipetted in water and sodium dodecyl sulfate ACS reagent (SDS, 99.0%, Roth) and were boiled at 95°C for 5 min. The proteins were separated at 90 V for about 15 min and then at 110 V for further 60 min. Utilizing Trans-Blot^®^ Turbo™ Transfer System (BioRad) the proteins were transferred onto a nitrocellulose membrane and then blocked with 5% skim milk powder (Sigma) in Tris-buffered saline with 0.1% TWEEN-20 (TBST), followed by an overnight incubation with primary antibodies at 4°C. The following primary antibodies were used: anti-β-Actin (ms, 1: 250,000, Sigma A5316), anti-SHANK3 [rb, 1:500, homemade, (Schmeisser, 2012)], anti-Calsequestrin (ms, 1:500, Thermo Fisher MA3-913), anti-RyR (rb, 1:500, Abcam ab219798), anti-DHPR (ms, 1:500, Abcam ab2862), anti-PTK6 (ms, 1:500, Santa cruz sc-166171), anti-Cav1.3 (rb, 1:500, Invitrogen ab85491), anti-KCKN18 (rb, 1:500, ThermoFisher PA5-114308). After washing the membrane three times for 20 min with 0.1% TBST and then incubating with HRP-conjugated secondary antibodies (Dako) at RT for 1 h, the membrane was washed three times for 20 min with 0.1% TBST. Protein bands were detected using the Chemiluminescent Western blot Reagent (Thermo Fischer), and were examined utilizing (Gel-analyzer 2010) (http://www.gelanalyzer.com/download.html).

### 2.8 Expansion microscopy

The TREx procedure was applied to enlarge the 9 µm thick human muscle sections on super-frost plus slides from control and PMS patient samples, with minor modifications based on a previously published protocol (Damstra et al., 2022). Briefly, muscle sections were washed shortly with PBS−/− (Gibco) and coated with 10 μg/mL acryloyl X-SE in PBS^−/−^ overnight at RT. For polymerization, muscle slices were kept with gelation solution (1.1 M sodium acrylate, 2.0 M acrylamide, 50 ppm N,N′- methylenebisacrylamide, TEMED (1.5 ppt), APS [1.5 ppt) and PBS^−/−^ (Gibco)] in a humid gelation chamber for 1 h at 37°C. The sections were gently removed from the slides using a brush and incubated in digestion buffer (50 mM Tris-BASE, 200 mM NaCl, 200 mM SDS in ddH2O) for 4 h at 80°C in a Thermo-Block. The gel was then washed 3x with PBS −/− (Gibco) for 5 min each and blocked for 3 h in blocking solution (0.3% Triton-X-100% and 3% BSA in PBS−/− (Gibco)). The primary Antibodies (SHANK3 (rb, 1:200, Schmeisser, 2012), DHPR (ms, 1:200, Abcam), CSQ (ms, 1:200, Sigma) and RyR (ms, 1:200, ENZO), PTK6 (ms, 1:200, Santa cruz) were diluted in blocking solution and incubated over weekend on a shaker (Unimax 1010) at 4 °C. The samples were then washed 3 times for 30 min with PBS−/− (Gibco) on a shaker (Unimax 1010) at RT. Secondary antibodies (Alexa Fluor 488 donkey anti-rabbit, Alexa Fluor 594 donkey anti-rabbit, Alexa Fluor 488 donkey anti-mouse, Alexa Fluor 594 donkey anti-mouse, Jackson ImmunoResearch Laboratories) were diluted in blocking buffer and incubated overnight on a shaker (Unimax 1010) at RT. The samples were then washed 3 times for 30 min with PBS−/− (Gibco) 3x 30 min ODER overnight on a shaker (Unimax 1010) at RT. The gel was then rinsed once with PBS−/− (Gibco) for 5 min. The stained gel was then treated with DAPI (1:50.000, Roth) for 5 min oder overnight on a shaker (Unimax 1010) at RT. Following that, the samples were washed 4-5 times in ddH2O for 15 min and kept at 4°C overnight to enable the gel to fully expand. The region of interest was removed from the gel with a scalpel and put on a PLL covered #1.5H chambered cover glass to obtain images. Images were taken with a Leica TCS SPE II confocal microscope (Wetzlar, Germany) and processed further with FIJI (ImageJ).

### 2.9 Image analysis

Five electron microscopic images from three *Shank3(+/+)* and three *Shank3Δ11(−/−)* mice muscle sections were determined utilizing the “free hand selections” tool in Fiji (ImageJ) to measure the area of the sarcoplasmic reticulum.

Five confocal images per animal were analyzed for quantification of CSQ, RyR, DHPR, Cav1.3, KCKN18, and PTK6 in the gastrocnemius muscle by collapsing three slides at maximum projection and measuring the thresholded area, mean gray value, and total intensity of the positive area using Fiji ImageJ. For each image, experimental groups were imaged together with the corresponding control. Mice data were normalized to the mean of all *Shank3(+/+)* data results, while human data were normalized to the mean of respective control data points achieved from the same experiment. For the human investigation, three technical replicates were examined.

Five confocal images per condition were analyzed for quantification of SHANK3, RyR, DHPR, CSQ and CEPIA in the α-Actinin and GFP positive myotubes and measuring the mean gray value and the positive area in each myotube. Knock-down vs. Control analysis were normalized to the mean of both conditions. 4CmC treatment data were normalized to the mean of all Control vehicle data result.

### 2.10 Bioinformatic analyses of RNA-sequencing data

The gastrocnemius muscle of four P0-P1 *Shank3(+/+)* and five *Shank3Δ11(−/−)* mice were used for whole-transcriptome analysis, at which RNA extraction, library preparation, cluster generation and sequencing were executed at the Eurofins Genomics Germany GmbH. Initially, 1 ng/μL of RNA per sample was used for RNA sampling. With the same primers, the sequences of (*Shank3Δ11(−/−)*) mice were group-wise compared to sequences obtained from *Shank3(+/+)* RNA. However, due to insufficient number of total reads, two *Shank3Δ11(−/−)* (KO 2 and 4) were excluded from the subsequent data analysis (Supplementary Figures S1A, B). Raw reads were mapped to the UCSC mouse genome version 10 (mm10) using HISAT2 (Kim et al., 2015; 2019; Pertea et al., 2016; http://daehwankimlab.github.io/hisat2/; version 2.2.1). Concordantly mapped reads were kept for downstream analysis and gene counting was performed using featureCounts (Liao et al., 2014; https://subread.sourceforge.net/; version 2.0.1). Data normalization and differential expression analysis were performed using the R package limma (Ritchie et al., 2015; https://bioconductor.org/packages/release/bioc/html/limma.html; version 3.56.2). Heatmaps, Principal Component Analysis (PCA), and radar plots were also generated using R (Free Software Foundation, 2015; https://cran.r-project.org; version 3.6). Gene Set (Enrichment Analysis GSEA) was performed using the standalone GSEA java application (Mootha et al., 2003; Subramanian et al., 2005; https://www.gsea-msigdb.org/gsea/index.jsp; GSEA version 4.3.2 and MSigDB version 7.1).

### 2.11 Statistical analysis

GraphPad Prism 8 (GraphPad Software Inc., 2022) and/or Microsoft Excel (Microsoft, 2016) were used for statistical analysis of data and graphs. The mean ± Standard Error of Mean (SEM) is displayed in the data. The Shapiro-Wilk normality test or D’Agostino–Pearson test was used to investigate data for normality. To compare two groups, the two-tailed Student's t-test was used, while one-way analysis of variance (ANOVA) with Tukey´s *post hoc* analysis was applied to compare more than two groups. The significance levels (*p* values) were set to 0.05 (*p* 0.05*, *p* 0.01**, *p* 0.001***, *p* 0.001****) with a 95% confidence interval.

## 3 Results

### 3.1 Striated muscles of newborn *Shank3Δ11(−/−)* mice display an enlarged sarcoplasmic reticulum (SR) and intracellular calcium-ion upregulation

**FIGURE 1:**
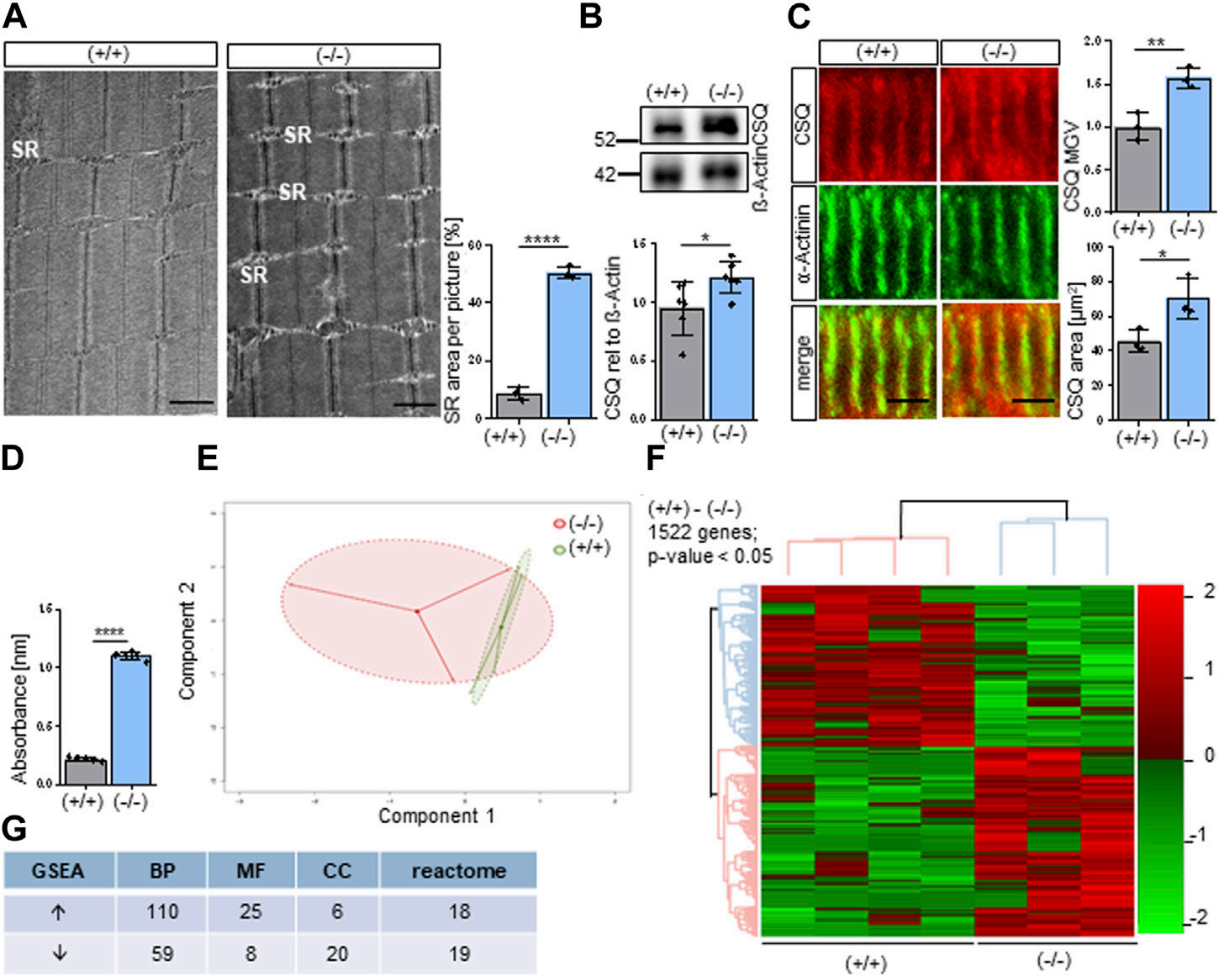
Altered molecular composition of the Sarcoplasmic Reticulum (SR) in *Shank3Δ11(−/−)* mice. **(A)** TEM analysis of the sarcoplasmic reticulum in P0-1 *Shank3Δ11(−/−)* and *Shank3(+/+)* animals. *n* = 3 animals per genotype. Means ± SEM, Student’s Unpaired *t*-test, *p* = 0.0001. Scale bar 1 μm. **(B)** Western blot analysis of CSQ and ß-Actin in skeletal muscle tissue lysates. *n* = 6 animals per genotype. Means ± SEM, Student’s Unpaired *t*-test, *p* = 0.0370. **(C)** Representative confocal images and quantification indicate CSQ intensity in skeletal muscle sections in P0-1 *Shank3Δ11(−/−)* mice. *n* = 3 animals per genotype. Means ± SEM, Student’s Unpaired *t*-test, area *p* = 0.0663, MGV *p* = 0.0257. Scale bar 10 μm. **(D)** BAPTA absorbance at 310 nm indicating total calcium concentration in the skeletal muscle. *n* = 5 animals per genotype. Means ± SEM, Student’s t test, BAPTA *p* = 0.0001. **(E)** Principal component analysis of transcriptomic data from three *Shank3Δ11(−/−)* and four *Shank3(+/+)* animals. Each dot represents a biological replicate. **(F)** Heat map of DEGs in P0-1 *Shank3Δ11(−/−)* vs. *Shank3(+/+)* mice transcriptomes. Color scale illustrate gene expression relative to *Shank3(+/+).* **(G)** Amount of identified up and downregulated gene sets of the three gene ontology domains: biological processes (BP), molecular function (MF), cellular components (CC) and reactome. SR, sarcoplasmic reticulum; BAPTA: 1,2-bis(o-aminophenoxy)ethane-N,N,N′,N′-tetraacetic acid), CSQ, calsequestrin; DEG, Differentially Expressed Gene; MGV, Mean Gray Value; SEM, Standard Error of the Mean; TEM, Transmission electron microscopy.

Since we obtained enlarged SR in adult *Shank3Δ11(−/−)* mice (Lutz et al., 2020) and interpreted this as a putative compensatory mechanism in Phelan-McDermid Syndrome (PMS) we focused on structural alterations in muscles of newborn mice (P0-P1). Unexpectedly, we confirmed an enlargement of the SR in neonatal *Shank3Δ11(−/−)* mice as revealed by EM analysis (Figure 1A). Next, we confirmed these data by the immunostainings of SR calcium-binding protein calsequestrin (CSQ). We obtained increased CSQ levels in Western blot and immunostainings of P0-1 *Shank3Δ11(−/−)* muscle tissue and observed increased CSQ accumulation (Supplementary Figure S1C), verifying the widening of the SR (Figures 1B, C). Next, we determined intracellular calcium-levels using the calcium chelators BAPTA (Figure 1D) and EDTA (Supplementary Figure S1D) and measured significantly increased calcium absorbance in the *Shank3Δ11(−/−)* compared to *Shank3(+/+)* mice.

In a screening approach we performed RNA sequencing of the gastrocnemius muscle of neonatal P0-P1 *Shank3(+/+)* and *Shank3Δ11(−/−)* mice. Principal Component Analysis (PCA) revealed a distinct clustering of the *Shank3Δ11(−/−)* results from the *Shank3(+/+)*, however, the *Shank3Δ11(−/−)* animals showed higher variation (Figure 1E). We identified 1522 differentially expressed genes (DEGs) in the *Shank3Δ11(−/−)* compared to the *Shank3(+/+)* skeletal muscle with a *p*-value <0.05 and FDR = 25% (Figure 1F). The top 20 up and downregulated DEGs for non-protein coding transcript variants and the top 10 protein-coding genes are summarized in Supplementary Table S1A, 2A. Using Gene Set Enrichment Analysis (GSEA) of Gene Ontology (GO) terms, we identified 110 and 59 gene sets were up and downregulated in the GO category Biological Processes (BP), respectively (Figure 1G). A comparison of the *Shank3(+/+)* and *Shank3Δ11(−/−)* data sets for the GO-term Molecular Function (MF) revealed 25 upregulated and 8 downregulated gene set. For the GO-term Cellular Component (CC), 6 gene sets were upregulated while 20 were downregulated. Additionally, a REACTOME GSEA revealed 18 upregulated and 19 downregulated gene sets (Figure 1G).

### 3.2 Neonatal *Shank3Δ11(−/−)* skeletal muscle transcriptome analysis reveals dysregulated calcium homeostasis pathways

**FIGURE 2:**
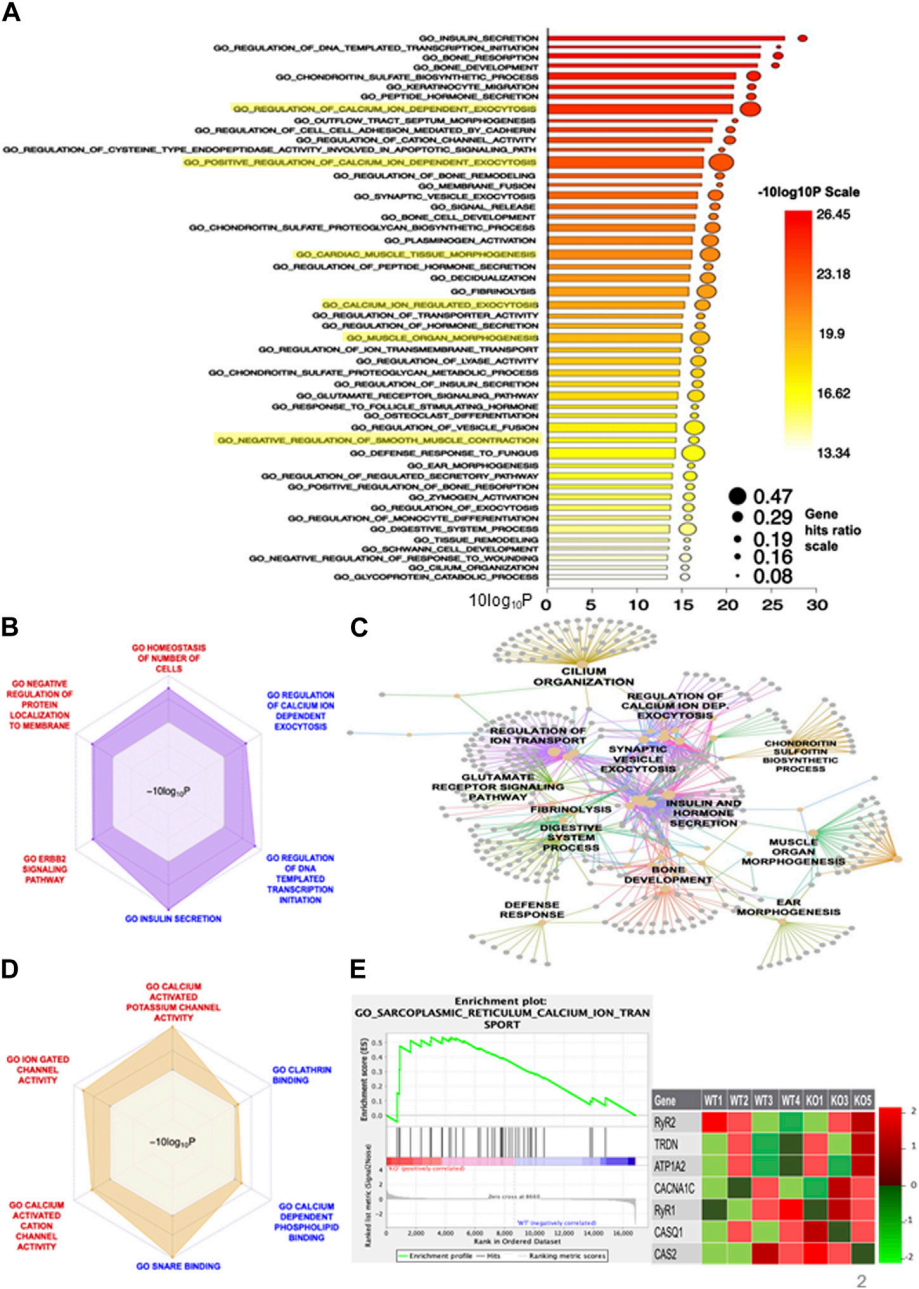
RNA-Sequencing reveals a modified transcriptome in *Shank3Δ11(−/−)* mice muscle. **(A)** Barplot of GO Biological Processes of the significantly altered pathways. **(B)** Radarplot representing selected up- or downregulated BP cluster. **(C)** Visualization of the network between the DEGs of the BP pathway and the amount of genes involved. **(D)** Up-and downregulated pathways of the GO term Molecular Function. **(E)** The increased expression of transcripts related to “SR calcium ion transport” is confirmed by the enrichment blot and the list of selected core genes displayed. Supplementary Table S2B shows the lists of all core genes. BP, Biological Processes; DEG, Differentially Expressed Gene, Gene Set (Enrichment Analysis GSEA), GO, Gene Ontology; SR, Sarcoplasmic reticulum.

According to the aforementioned findings, *Shank3Δ11(−/−)* show global transcriptional change in comparison to *Shank3(+/+)*. The analysis of the GO category BP revealed enrichment of the term “Calcium ion dependent exocytosis,” with 47% of genes in this process were deregulated. Furthermore, the terms “regulation of calcium ion dependent exocytosis” (29% of genes in the term) and “Calcium ion regulated exocytosis” (19% of genes in the set) showed significant enrichment. Moreover, the GO analysis revealed modifications linked to “cardiac muscle tissue morphogenesis” (29% of genes), “muscle organ morphogenesis” (29% of genes) and “negative regulation of smooth muscle contraction” (16% of genes) (Figure 2A), highlighted in yellow. These findings imply that hypotonia might be attributed to different biological processes with a special focus on calcium -related alterations. Selected significantly upregulated (“regulation of calcium ion dependent exocytosis,” “DNA template transcription initiation and “Insulin secretion”) and downregulated (“homeostasis of number of cells,” “negative regulation of protein localization to membrane” and “ERBB2 signaling pathway”) of the GO BP are presented using radar plot in Figure 2B. These results indicate that SHANK3 loss has a significant impact on a variety of biological functions. To determine how those biological processes are connected, we performed an interaction network analysis and found that a core cluster is formed between “Regulation of calcium ion dependent exocytosis,” “Synaptic vesicle exocytosis” and “Regulation of ion transport,” again highlighting the central role of calcium (Figure 2C). Similarly, GO molecular function-based radar plot using selected calcium-related gene sets showed upregulation of “Calcium dependent phospholipid binding” and downregulation in “Calcium activated potassium channel activity,” “Ion gated channel activity” and “Calcium activated cation channel activity” (Figure 2D). These findings show how Shank3 can impact calcium-related functions in mouse skeletal muscle cells. Third, the radar plot of the GO cellular component revealed significantly downregulated gene sets involved in “soluble N-ethylmaleimide sensitive factor attachment protein receptors (SNARE) complex” and “postsynaptic membrane” (Supplementary Figure S1E), which suggests a potential impact on cellular processes related to synaptic transmission. Finally, the GO term REACTOME revealed “Signaling by PTK6” as one of the upregulated gene sets (Supplementary Figure S1F) that caught our interest, since PTK6 is involved in ERBB2 signaling pathway (Xiang et al., 2008) and transcriptional regulation (Harvey and Burmi, 2011). We used GSEA to compare our data to the previously published “Sarcoplasmic reticulum calcium ion transport” datasets. Interestingly, we found 37 SR-related genes to be significantly upregulated in the *Shank3Δ11(−/−)* mice (Figure 2E), including ryanodine receptors (RyR) that releases calcium ions from the sarcoplasmic reticulum into the cytosol, calcium binding protein calsequestrin and voltage-gated calcium channel to maintain the muscle contraction.

### 3.3 Receptors of the sarcolemma are altered in *Shank3Δ11(−/−)* muscle

**FIGURE 3:**
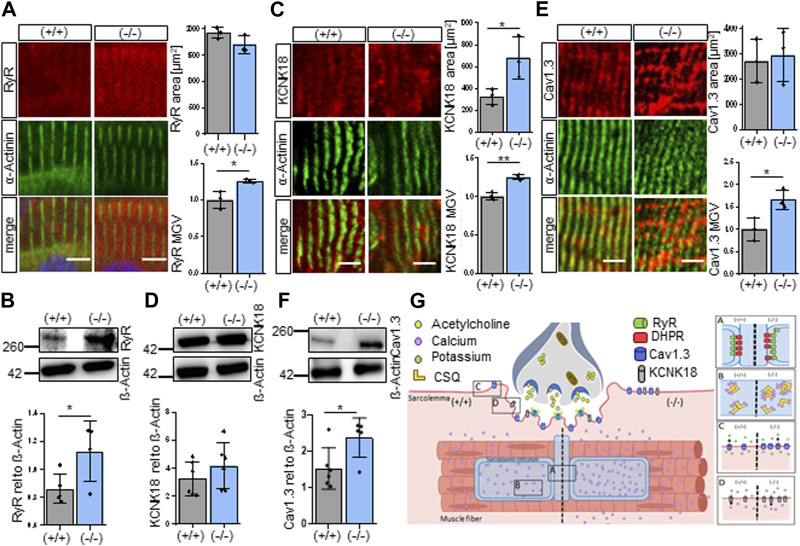
*Shank3Δ11(−/−)* mice demonstrate increased expression of sarcolemmal receptors in skeletal muscle sections. **(A)** Immunofluorescence images of skeletal muscle tissue of P0-1 *Shank3Δ11(−/−)* and *Shank3(+/+)* mice for RyR (red) and α-Actinin (green). *n* = 3 animals per genotype. Means ± SEM, Student’s Unpaired *t*-test, area *p* = 0.4038, MGV *p* = 0.2488. Scale bar 10 μm. **(B)** Western blot analysis of RyR and ß-Actin in skeletal muscle tissue lysates. *n* = 6 animals per genotype. Means ± SEM, Student’s Unpaired *t*-test, *p* = 0.0404. **(C)** Immunofluorescence images of skeletal muscle tissue of P0-1 *Shank3Δ11(−/−)* and *Shank3(+/+)* mice for KCNK18 (red) and α-Actinin (green). *n* = 3 animals per genotype. Means ± SEM, Student’s Unpaired *t*-test, area *p* = 0.0396, MGV *p* = 0.0023. Scale bar 5 μm. **(D)** Western blot analysis of KCNK18 and ß-Actin in skeletal muscle tissue lysates. *n* = 6 animals per genotype. Means ± SEM, Student’s Unpaired *t*-test, *p* = 0.2932. **(E)** Immunofluorescence images of skeletal muscle tissue of P0-1 *Shank3Δ11(−/−)* and *Shank3(+/+)* mice for Cav1.3 (red) and α-Actinin (green). *n* = 3 animals per genotype. Means ± SEM, Student’s Unpaired *t*-test, area *p* = 0.7806, MGV *p* = 0.0261. Scale bar 5 μm. **(F)** Western blot analysis of Cav1.3 and ß-Actin in skeletal muscle tissue lysates. *n* = 6 animals per genotype. Means ± SEM, Student’s Unpaired *t*-test, *p* = 0.0318. **(G)** Illustration of the SHANK3 associated alterations in the receptor expression and calcium dysregulation on the sarcolemma of *Shank3Δ11(−/−)* mice (right panel) compared to *Shank3(+/+)* (left panel), matched with the RNA-sequencing results. Cav1.3: Voltage-gated calcium channel, KCNK18: Calcium-gated potassium channel, MGV, Mean Gray Value; RyR, Ryanodine receptor; SEM, Standard Error of the Mean.

To elucidate if the findings of our RNA sequencing results also translated to changes in protein expression, we examined specific proteins that were selected from the GO “Sarcoplasmic reticulum calcium ion transport” and that contributed in addition to muscular contraction and might explain the occurrence of hypotonia. The genes DHPR, RyR, CACNA1D, KCNK18 and CSQ are functionally important for the calcium homeostasis leading to muscle contraction and were found to be significantly upregulated in the *Shank3Δ11(−/−)* mice. Now, the proteins of these genes were examined in the skeletal muscle of P0-P1 mice by immunostaining or Western blotting. Indeed, the RyR, the calcium channel that permits calcium from the SR to enter into the cytoplasm, was altered. Total RyR protein expression was increased in line with the increased gene expression (Figures 3A, B). Additionally, the *Shank3Δ11(−/−)* mice exhibit smaller area, relative frequency size and RyR particle sizes (Figure 3A; Supplementary Figure S2A), indicating that despite a higher total RyR amount the functional units are smaller than in the *Shank3(+/+)*, suggesting a different morphological arrangement of the RyR complex. To follow this hypothesis, we also examined the intensity as Mean Gray Value (MGV) and the area of the Dihydropyridine receptor (DHPR), that is structurally coupled to RyR functioning as its calcium sensor for opening the channel and allowing the influx of calcium ions into the muscle cell. However, we did not find any changes (Supplementary Figures S2B, C). We then investigated whether SHANK3 deficiency affects the Calcium-gated potassium channel (KCNK18) expression that is involved in muscle excitability and contractibility, as indicated by the transcriptomic analysis. Indeed, we confirmed the upregulation of KCNK18 area and intensity with immunofluorescence but not in Western blot (Figures 3C, D). We further assessed the area and intensity of the voltage-gated calcium channel (Cav1.3), since it is important for the initiation and maintenance of muscle contraction by allowing the influx of calcium ions into the muscle cell. In agreement with the RNA-Seq results we observed an elevated Cav1.3 expressions in *Shank3Δ11(−/−)* mice compared to *Shank3(+/+)* (Figures 3E, F). *Shank3Δ11(−/−)* mice revealed unchanged PTK6 area, but in contrast an increased intensity (Supplementary Figures S2D, E).

Taken together, in P0-1 *Shank3Δ11(−/−)* mice we found changes in the protein expression of DHPR, RyR, CACNA1D, KCNK18 and CSQ that all contribute to the maintenance of calcium homeostasis (Figure 3G). We found the total RyR amount to be increased but in smaller functional units [Figure 3G-(A)] together with an increased expression of CSQ [Figure 3G-(B)], Cav1.3 [Figure 3G-(C)] and KCNK18 [Figure 3G-(D)] which are all involved in muscle excitability and contractibility. On the other hand, there were no changes found in the expression of the Dihydropyridine receptor (A). These findings suggest that SHANK3 deficiency leads to a significant misregulation of calcium homeostasis which may contribute to the development of hypotonia in *Shank3Δ11(−/−)* mice.

### 3.4 Skeletal muscle biopsies of patients with PMS display calcium misregulation and changes in sarcolemmal receptors

**FIGURE 4:**
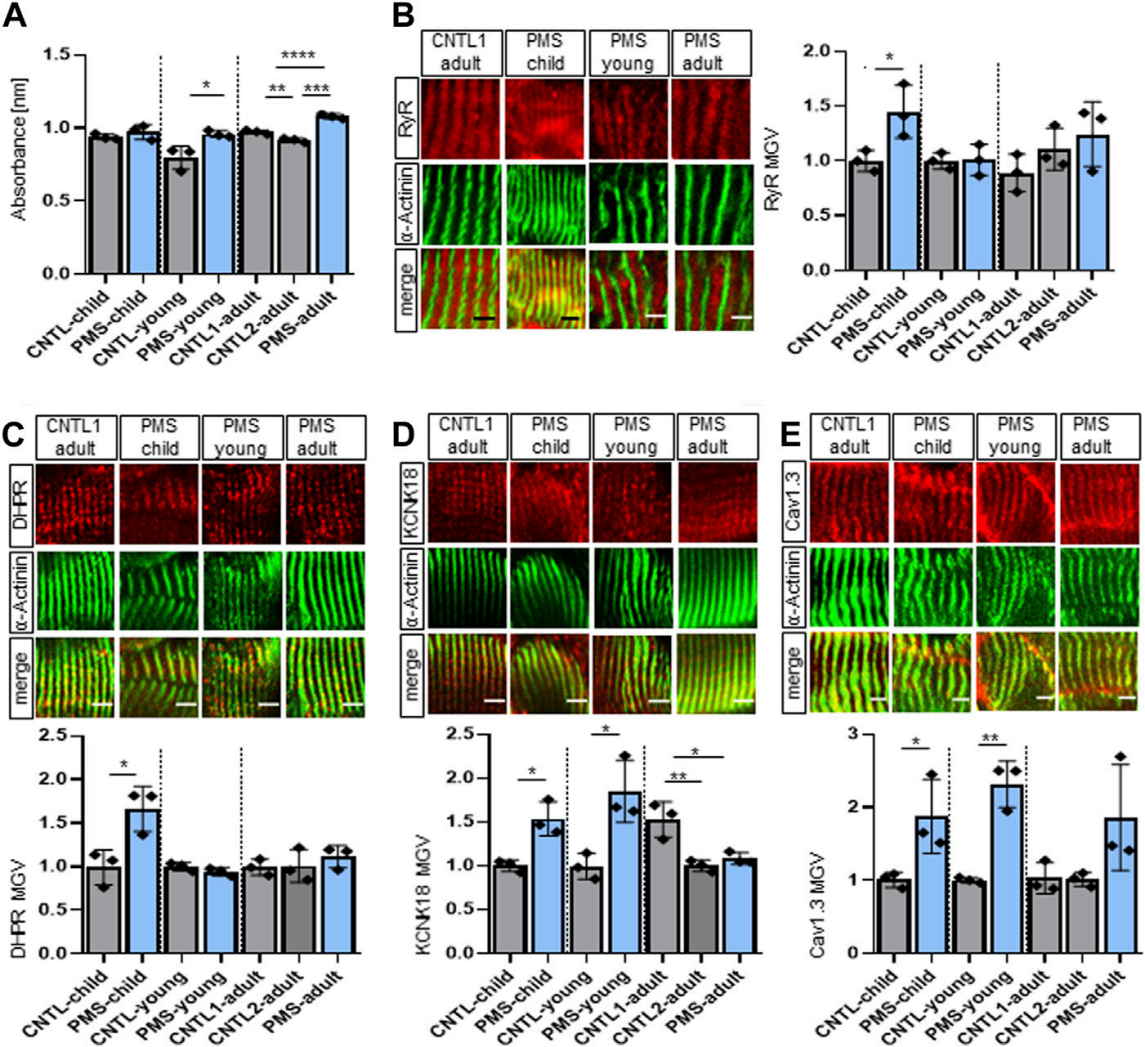
Changed receptor expression in muscle biopsies of patients with PMS. **(A)** BAPTA absorbance at 310 nm indicating total calcium concentration in the skeletal muscle. *n* equals number of technical replicates. Means ± SEM, Student’s *t* test for child and young and one-way ANOVA and Tukey’s multiple comparisons test for adult, child: *p* = 0.3579, young: *p* = 0.0272 and adult C1 vs. C2: *p* = 0.0086, C1 vs. PMS: *p* = 0.0003 and C2 vs. PMS: *p* < 0.0001. **(B)** Immunofluorescence images of skeletal muscle tissue of human biopsies for RyR (red) and α-Actinin (green). *n* equals number of technical replicates. Means ± SEM, Student’s Unpaired *t*-test for child and young and one-way ANOVA and Tukey’s multiple comparisons test for adult, RyR MGV child: *p* = 0.0395, young: *p* = 0.9291 and adult C1 vs. C2: *p* = 0.5056, C1 vs. PMS: *p* = 0.2161 and C2 vs. PMS: *p* = 0.7561. Scale bar 5 μm. **(C)** Immunofluorescence images of skeletal muscle tissue of human biopsies for DHPR (red) and α-Actinin (green). *n* equals number of technical replicates. Means ± SEM, Student’s Unpaired *t*-test for child and young and one-way ANOVA and Tukey’s multiple comparisons test for adult, DHPR MGV child: *p* = 0.0232, young: *p* = 0.2016 and adult C1 vs. C2: *p* = 0.9903, C1 vs. PMS: *p* = 0.5853 and C2 vs. PMS: *p* = 0.6601. Scale bar 5 μm. **(D)** Immunofluorescence images of skeletal muscle tissue of human biopsies for KCNK18 (red) and α-Actinin (green). *n* equals number of technical replicates. Means ± SEM, Student’s Unpaired *t*-test for child and young and one-way ANOVA and Tukey’s multiple comparisons test for adult, KCNK18 MGV child: *p* = 0.0108, young: *p* = 0.0180 and adult C1 vs. C2: *p* = 0.0058, C1 vs. PMS: *p* = 0.0130 and C2 vs. PMS: *p* = 0.7261. Scale bar 5 μm. **(E)** Immunofluorescence images of skeletal muscle tissue of human biopsies for Cav1.3 (red) and α-Actinin (green). *n* equals number of technical replicates. Means ± SEM, Student’s Unpaired *t*-test for child and young and one-way ANOVA and Tukey’s multiple comparisons test for adult, Cav1.3 MGV child: *p* = 0.0438, young: *p* = 0.0021 and adult C1 vs. C2: *p* = 0.9986, C1 vs. PMS: *p* = 0.1306 and C2 vs. PMS: *p* = 0.1225. Scale bar 5 μm. BAPTA: 1,2-bis(o-aminophenoxy) ethane-N,N,N′,N′-tetraacetic acid), Cav1.3, Voltage-gated calcium channel; CSQ, calsequestrin; KCNK18, Calcium-gated potassium channel; MGV, Mean Gray Value; RyR, Ryanodine receptor; SEM, Standard Error of the Mean.

Since we found an increased calcium amount and changes in receptors associated with the sarcolemma in *Shank3Δ11(−/−)* mice, we wondered if similar changes could be found in patient-derived material. For this, the same PMS patients have been analyzed as in a previous study (Lutz et al., 2020). Young and adult PMS patients revealed an increased calcium absorbance using calcium-chelators BAPTA (Figure 4A) and EDTA (Supplementary Figure S3A), while this impact was not detected in the child PMS patient. To gain further insight into the molecular changes, we examined the same proteins as in mouse tissue RyR, DHPR, KCNK18, Cav1.3, and PTK6.

We saw an increase in the RyR amount, but only in the child PMS patient (Figure 4B), which nicely relates to the changes in newborn P0-1 *Shank3Δ11(−/−)* mice. The RyR area remained unchanged for all PMS patients (Supplementary Figure S3B).

In contrast to the mouse data, the DHPR was also affected in PMS patients. The area as well as the total DHPR intensity were decreased in all PMS patients compared to their respective controls, in contrast, the DHPR MGV was increased only in the child (Figure 4C; Supplementary Figure S3C). Interestingly, the KCNK18 intensity was only increased in the child and young PMS patients, but not in the adult (Figure 4D). While the KCNK18 area remained unchanged in the child and young PMS patient, it significantly increased in the adult PMS patient compared to their respective controls area (Supplementary Figure S3D), therefore altogether reporting changes in KCNK18 in all PMS samples of all ages. Regarding the calcium channel Cav1.3, the intensity was significantly increased in all PMS patients (Figure 4E). The Cav1.3 area was significantly increased only in young PMS patient, but not in child and adult (Supplementary Figure S3E) and the total intensity was increased in all patients. We found some intriguing results regarding PTK6 expression in PMS patients. The PTK6 area was elevated in all PMS patients. This finding is especially meaningful given that PTK6 was discovered to be one of the top 20 elevated genes in *Shank3Δ11(−/−)* mice. Moreover, while the intensity of PTK6 was downregulated in young and adult PMS patients, it remained unchanged in child (Supplementary Figure S3F). Furthermore, the total PTK6 intensity was on average increased in all PMS patients and demonstrated a moderate correlation with SHANK3 in expansion microscopy (Supplementary Figure S3F, Supplementary Figure S4D). Expansion microscopy of human skeletal muscle sections from control and PMS patients revealed no correlation between SHANK3 and RyR, SHANK3 and DHPR as well as SHANK3 and CSQ, indicating to an indirect effect of SHANK3 on the SR receptor membranes (Supplementary Figures S4A–C).

In conclusion, the analysis of patient-derived muscle biopsy from PMS patients showed increased calcium absorbance in the young and adult patients, compared to their respective controls. This suggests a dysregulation of calcium metabolism. Further evaluation of the proteins RyR, DHPR, KCNK18, Cav1.3, and PTK6, which are involved in calcium homeostasis and muscle contraction, showed calcium-store, -release and changes in transmembrane-protein expression in PMS patients compared to controls. These findings support the complexity of the misbalance in the regulation of calcium homeostasis present in PMS patients.

### 3.5 SHANK3 knock-down causes alterations in sarcolemmal receptors and the sarcoplasmic reticulum in C2C12-derived myotubes

**FIGURE 5:**
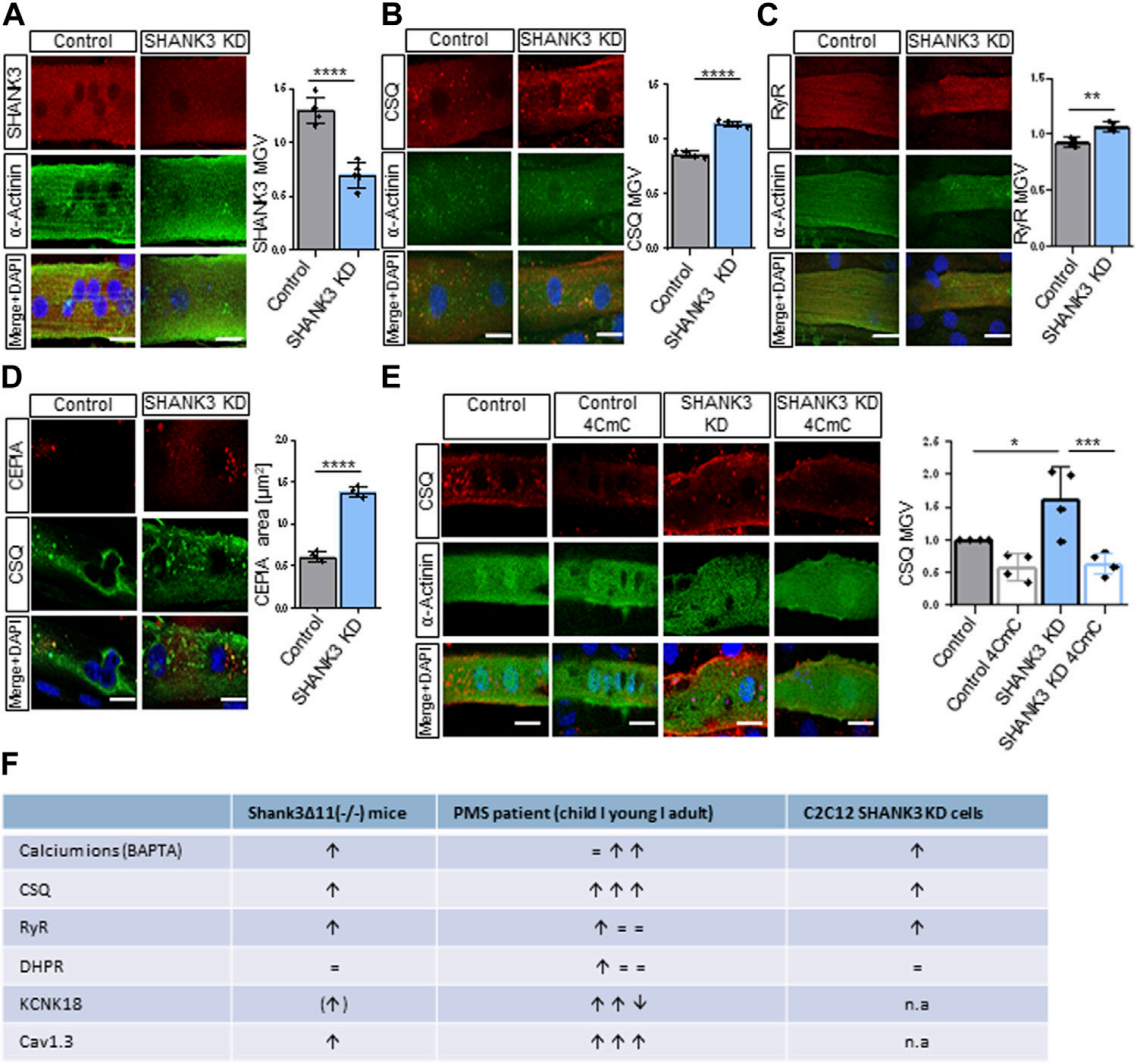
Sarcolemmal receptors and the sarcoplasmic reticulum in C2C12-derived myotubes are altered in SHANK3 knock-down. **(A)** Immunofluorescence images of transfected C2C12 myotubes for SHANK3 (red) and α-Actinin (green). *n =* 4 technical replicates. Means ± SEM, Student’s Unpaired *t*-test, SHANK3 MGV: *p* < 0.0001. Scale bar 50 μm. **(B)** Immunofluorescence images of transfected C2C12 myotubes for CSQ (red) and α-Actinin (green). *n* equals number of technical replicates. Means ± SEM, Student’s Unpaired *t*-test, CSQ MGV: *p* < 0.0001. Scale bar 50 μm. **(C)** Immunofluorescence images of transfected C2C12 myotubes for RyR (red) and α-Actinin (green). *n* equals number of technical replicates. Means ± SEM, Student’s Unpaired *t*-test, RyR MGV: *p* = 0.0034. Scale bar 50 μm. **(D)** Immunofluorescence images of transfected C2C12 myotubes for CEPIA (red) and CSQ (green). *n* equals number of technical replicates. Means ± SEM, Student’s Unpaired *t*-test, CEPIA area: *p* < 0.0001. Scale bar 50 μm. **(E)** Immunofluorescence images of transfected C2C12 myotubes treatment with or without 4CmC for CSQ (red). *n* equals number of technical replicates. Means ± SEM, Two-way-ANOVA, MGV: *p* < 0.0001. Scale bar 50 μm. Two-way ANOVA, interaction ns, genotype *p* = 0.0336, treatment *p* = 0.0003. Bonferroni’s multiple comparisons test, KD vehicle vs. KD 4-CMC *p* = 0.0007, Control vehicle vs. KD vehicle *p* = 0.0182. **(F)** Summary of Calcium Dysregulation in *Shank3Δ11(−/−)* mice, PMS patients and C2C12 SHANK3 KD cells. 4CmC, 4-Chloro-m-cresol; CEPIA, Calcium-measuring organelle-entrapped protein indicator; CSQ, Calsequestrin; KD, Knock-down; MGV, Mean Gray Value; n.a, not assessed; RyR, Ryanodine receptor; SEM, Standard Error of the Mean.

To investigate if a loss of SHANK3 in muscle cells can directly lead to the alterations we observed *in vivo*, myotubes derived from the well-established myoblast cell line C2C12 were used as an *in vitro* model. Cells were transfected with GFP (control) or SHANK3 siRNA to induce a knock-down (KD) (Supplementary Figure S5A). Only GFP-positive cells were subsequently analyzed. The SHANK3 intensity was significantly decreased in SHANK3 KD (Figure 5A). Comparable to mice skeletal muscle data (Figure 1C, Figure 3A), CSQ (Figure 5B) and RyR intensity (Figure 5C) were significantly increased, suggesting a dysregulation of SR calcium storage and release mechanism caused by a SHANK3 deficit. Similar to the skeletal mouse results, the DHPR intensity was not altered (Supplementary Figure S5B). To monitor calcium level found solely in SR of mice myocytes we used the genetically encoded fluorescent calcium indicator Calcium-measuring organelle-entrapped protein indicator (CEPIA). We obtained an increased CEPIA area in the SHANK3 KD, that corresponds to the enlargement of the SR (Figure 5D). However, the intensity was unchanged (Supplementary Figure S5C). These results indicate that there is a direct link between SHANK3 levels in a muscle cell and the regulation of calcium storage. The chemical compound 4-Chloro-m-cresol (4CmC) is known for its function as a ryanodine receptor activator and sarcoplasmic-endoplasmic reticulum Calcium-ATPase (SERCA) inhibitor (Zorzato et al., 1993; Al-Mousa and Michelangeli, 2009). We aimed to study if 4CmC could lead to calcium sequestration of the SR to restore normal CSQ levels. Therefore, we applied 4CmC to differentiated C2C12 myotubes and compared the results to a vehicle control group. Indeed, 4CmC restored the intensity of CSQ in SHANK3 KD (Figure 5E), which confirms that a restoration of calcium equilibrium between SR and the sarcoplasma can reverse dysregulated protein expression in SHANK3 deficiency. We summarized the changes observed in skeletal muscle under SHANK3 loss in *Shank3Δ11(−/−)* mice, PMS patients, and C2C12 SHANK3 KD cells (Figure 5F).

## 4 Discussion

In this study we demonstrated that a Shank3 deletion in striated muscle cells leads to an enlarged Sarcoplasmic Reticulum (SR) and raises the levels of calcium and the calcium-binding protein calsequestrin (CSQ). Furthermore, utilizing RNA-sequencing of muscle tissue of newborn *Shank3Δ11(−/−)* mice, we showed that genes involved in calcium ion regulation and ion channels required for muscular contraction were affected by Shank3 loss. These alterations also translated into protein changes of calcium secretion proteins and calcium-related signaling proteins.

SHANK3 deficiency is known to be associated with muscular deficits (Zhou et al., 2019; Lutz et al., 2020; Bauer et al., 2023). However, the pathways by which a Shank3 deletion causes these effects and whether they result in hypotonia remain unknown. Based on skeletal muscle alterations in P56 *Shank3Δ11(−/−)* mice we hypothesize that the structural differences are due to compensatory mechanisms to rescue less functional contraction units due to SHANK3 loss (Lutz et al., 2020).

Interestingly, however, we discovered that the muscular phenotype is already present at very early stage of life by analyzing P0-P1 pups whose muscle coordination has not received sensory input. An important finding of the present study is the observed enlargement of the SR and increased CSQ levels in newborn P0-P1 *Shank3Δ11(−/−)* mice that were comparable to what we discovered in the P56 *Shank3Δ11(−/−)* mice and PMS patients (Lutz et al., 2020). A similar hallmark has been observed in patients with myotubular myopathy (Amoasii et al., 2013) that may lead to skeletal muscle weakness, comparable to patients with PMS (Phelan et al., 2001). As the primary calcium store, absorption and release site, the SR is essential for maintaining calcium levels, muscular contraction, and the positioning of muscle compartments (Luff and Atwood, 1971; Franzini-Armstrong, 1991; Pozzan et al., 1994; Takekura et al., 2001; Rossi et al., 2008). Comparable to our results, Rossi et al. displayed a correlation between abnormal T-tubule and SR structure with impaired calcium release and excitation–contraction coupling (Rossi et al., 2021). To investigate the context of the SR expansion, a transcriptome analysis of the skeletal muscle tissue of newborn *Shank3Δ11(−/−)* mice was performed. Indeed, our findings showed that SHANK3 is an important player in the regulation of calcium ion dependent exocytosis and muscle morphogenesis. We conducted an interaction network analysis to discover the relationships between those biological processes and revealed that “Regulation of calcium ion dependent exocytosis,” “Synaptic vesicle exocytosis,” and “Regulation of ion transport” form a core cluster, once more emphasizing the crucial role of SHANK3 on calcium homeostasis. Using Gene Set Enrichment Analysis (GSEA), we found that the “Sarcoplasmic reticulum calcium ion transport” gene set had a considerable enrichment of upregulated SR-related genes in the *Shank3Δ11(−/−)* mice. SHANK3 deficiency has been shown to result in increased intracellular calcium levels in neurons of the Shank3 R1117X+/+ mice (Ali et al., 2021). Mutations or aberrations in only one of the multiple proteins involved in intracellular calcium homeostasis can cause a variety of disorders (Missiaen et al., 2000; Lee et al., 2012; Limpitikul et al., 2016; Fill and Gillespie, 2021) and can potentially speed the onset of muscle diseases (Zheng et al., 2022). De Bartolomeis et al. demonstrated that scaffolding proteins like Homer, SHANK3, PSD95 can modulate calcium-dependent pathways (De Bartolomeis and Tomasetti, 2012).

We found that SHANK3 deficiency increased CSQ levels and caused molecular alterations in calcium storage and we assume an impairment in the calcium release system in muscle cells. CSQ regulates calcium release during muscle contraction (Yamaguchi and Kasai, 1998). The ability to store calcium in the SR is increased in mice overexpressing CSQ; however, due to a reduction in the density of L-type calcium channel currents and a delay of the kinetics of those channels’ activation, it resulted in fewer calcium sparks (Jones, 1998; Sato et al., 1998; Suzuki et al., 1999). Besides keeping the large amount of calcium necessary for muscle contraction in close proximity of Ryanodine receptors (RyR), CSQ acts as a calcium-sensor and regulates directly and/or indirectly channel opening, activity and organization (Györke et al., 2004; Flucher, 2020; Rossi et al., 2021). Despite clear structural distinctions between skeletal and heart muscles, many protein complexes are present in both groups. The deletion of Shank3 may disrupt these protein complexes, including the CSQ-Triadin-Junctin-RyR complex (Li et al., 2015), and the PLCß1b-Shank3-Homer-RyR complex (Grubb et al., 2012; Woodcock and Grubb, 2015), which are known to affect calcium concentration in cardiomyocytes (Grubb et al., 2012). The CSQ-Triadin-RyR-Homer complex, located in the SR of muscle cells, is essential for precise regulation of calcium release during muscle contraction (Li et al., 2015). Upon receiving a signal to contract, the complex plays a critical role in sensing and releasing calcium ions through RyR channels in response to the action potential, triggered by interactions with CSQ and Triadin. The SH3 domain of Shank3 interacts with the proline-rich sequences in Homer, forming a complex that influences the enzymatic activity of PLCβ1b. This modulation of PLCβ1b by the Homer-Shank3 complex can result in changes to downstream calcium signaling pathways and altered calcium release from intracellular stores, impacting cellular functions regulated by calcium dynamics (Grubb et al., 2012). Shank3 may act as scaffold protein that stabilizes the connection between the channels on the sarcolemma or the SR membrane by interacting with various proteins via its complex domains (Boeckers et al., 1999; Olson et al., 2005; Woodcock and Grubb, 2015). Therefore, an absence of Shank3 would have an effect both on the sarcolemmal and SR channels and might result in altered localization and functioning, that may lead to dysregulation of muscle contraction. Muscle contraction can be targeted by Tirasemtiv, a troponin activator that sensitizes troponin to calcium. Tirasemtiv treatment resulted in a prolonged wire hanging time, indicating improved muscular grip strength in *Shank3Δ11(−/−)* mice (Lutz et al., 2020). Lower calcium spark frequency is associated with smaller RyR cluster size (Galice et al., 2018). We documented smaller RyR cluster size in *Shank3Δ11(−/−)* mice that could result in fewer calcium sparks. Through Tirasemtiv, troponin is sensibilized to the limited quantity of calcium dispersed in the cytoplasm in the muscle, resulting in appropriate muscle strength (Lutz et al., 2020). Our findings revealed that 4CmC restored the intensity of CSQ in *in vitro* study of SHANK3 KD, suggesting that CSQ levels are influenced by calcium depletion of the SR and that this effect may be reversed by restoring calcium equilibrium.

In P0-P1 *Shank3Δ11(−/−)* mice, we noticed enhanced transcription of the voltage-gated calcium channels (Cav1.3), which was also confirmed at protein level. Studies have shown that SHANK3 interacts directly with Cav1.3 (Zhang et al., 2005; Gao et al., 2022), and regulate their localization, channel current and gene expression (De Bartolomeis and Tomasetti, 2012; Pym et al., 2017; Wang et al., 2017). Pym et al. (2017) reported diminished Ca_V_ calcium current in shn-1 mutant *C. elegans*, wherein enhanced Cav1.3 activities is associated with ASD (Pinggera and Striessnig, 2016). The C-term of Cav1.3 can operate as transcription factor in the nucleus and regulate the function of calcium-activated potassium channel that might explain the elevated transcription of these channels (Lu et al., 2015; Pym et al., 2017). SHANK3 deficiency lead to increased Phosphotyrosine kinase 6 (PTK6) expression. PTK6 is a non-receptor tyrosine kinase that is related to Src family protein kinases that can modulate transcription, RNA processing and differentiation (Harvey and Burmi, 2011). PTK6 consists of SH2, SH3 and kinase domain to interact with proteins with proline-rich region and function as scaffold molecule to stabilize signaling complexes (Harvey and Burmi, 2011; Qiu and Miller, 2004). During cell migration PTK6 interacts with scaffold proteins (Goel and Lukong, 2015). The interaction between Shank3 and PTK6 suggests their involvement in a scaffolding function (Harvey and Burmi, 2011), facilitating the interaction with novel targets related to calcium channel function and signaling. This scaffold formation may modulate signaling complexes and gene expression regulation via specific protein-protein binding domains, such as ankyrin repeats, SH3, PDZ, and SAM (sterile alpha motif). These interactions likely contribute to the modulation of calcium channel properties and downstream signaling pathways (De Bartolomeis and Tomasetti, 2012; Pym et al., 2017; Wang et al., 2017). Our RNA sequencing approach demonstrated that SHANK3 is involved in both scaffolding and transcriptional regulation. Although SHANK3 is not reported to be a transcription factor itself, it can interact with transcription factors and heterogeneous nuclear ribonucleoproteins, to regulate the transcription of genes (De Bartolomeis and Tomasetti, 2012; Grabrucker et al., 2014; Perfitt et al., 2020; Pym et al., 2017). Additionally, SHANK3 has been shown to interact with histone modifying enzymes and regulate the chromatin structure at gene regulatory regions, which can affect gene expression (Wang et al., 2020). The absence of SHANK3 led to an increase in the variation of gene expression profiles among samples, which suggest that the loss of function of these genes primarily affects gene expression in a more random manner, as described (Gauthier et al., 2010; Huppertz et al., 2021).

We hypothesize that SHANK3 haploinsufficiency impacts calcium modulation in muscle cells, which can result in an overall increase in intracellular calcium levels and thus have an impact on excitation-contraction-coupling and muscle strength, culminating in muscle weakness and hypotonia as reported in patients and animal models (Al-Qusairi et al., 2009; Dowling et al., 2009; Toussaint et al., 2011). The particular mechanisms by which SHANK3 accomplishes these results are unknown. Given that there is no direct interaction between SHANK3 and our proteins of interests (RyR, DHPR, KCNK18 and PTK6), it is possible that other pathways or mechanisms are involved. The Shank3Δ11 mice possess a targeted deletion that leads to decreased expression of the long SHANK3 isoforms. Despite this reduction, certain SHANK3 isoforms continue to be expressed (Lutz et al., 2020 and Lutz et al., 2022). Based on Bauer et al., 2023, where heterozygous and homozygous animals have been analyzed, this current study used *Shank3Δ11(−/−).* Using human muscle sections has limitations due to the small sample size of three patients, per age group one biopsy per individual, and the fact that they are separate individuals with genetic differences. Therefore, it is difficult to draw conclusions about long-term changes. Nevertheless, our study provides important insights into calcium dysregulation during aging, which may be conserved between humans and mice. Further studies with larger sample size are needed to confirm these findings and better understand the complex processes of calcium regulation in aging muscle.

SHANK3 has been shown to play a role in the regulation of muscle differentiation, gene expression, and muscle contraction, but the hypotonia is not only caused by a motor-neuron deficit, but also by muscle impairments and dysregulation of the SR constituents. This study contributes new insights into SHANK3 skeletal muscle pathology, and further highlights prospective targets for treatment of hypotonia in Phelan-McDermid syndrome.

## Data Availability

The data presented in the study are deposited in the Gene Expression Omnibus repository, accession number GSE241812. https://www.ncbi.nlm.nih.gov/geo/query/acc.cgi?acc=GSE241812.
