# Supplementary figures and images for: Shank3 related muscular hypotonia is accompanied by increased intracellular calcium concentrations and ion channel dysregulation in striated muscle tissue
Source: Front Cell Dev Biol. 2023 Sep 6;11:1243299. doi: 10.3389/fcell.2023.1243299 (PMC10511643; doi:10.3389/fcell.2023.1243299)

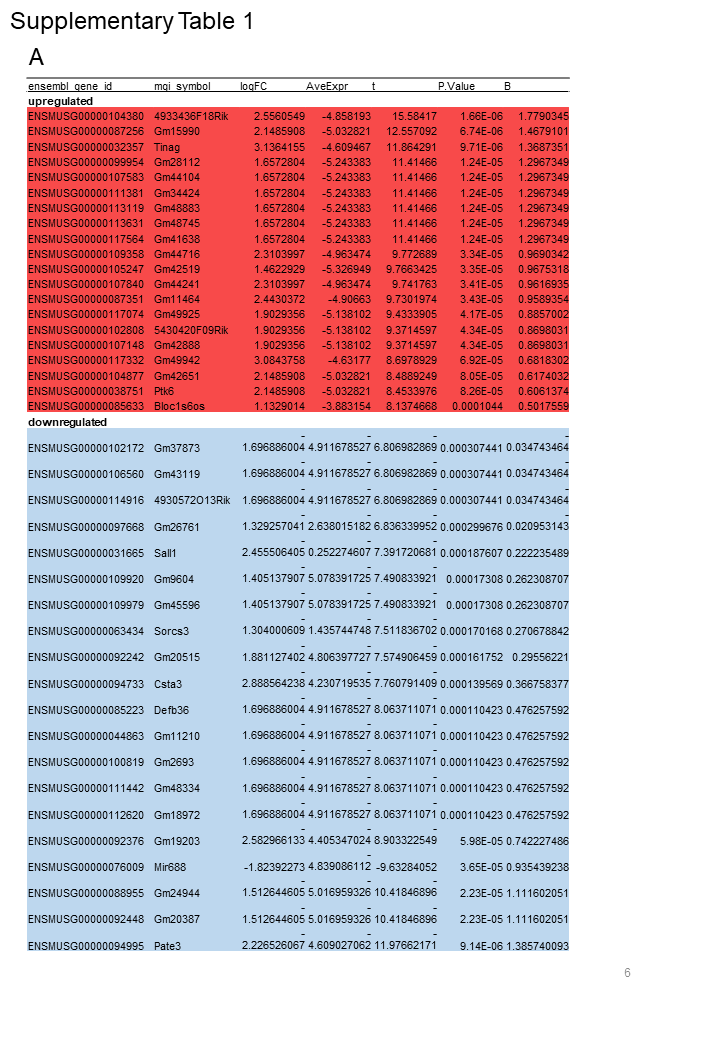

Supplement: Supplementary file 1 [file Image6.TIF]

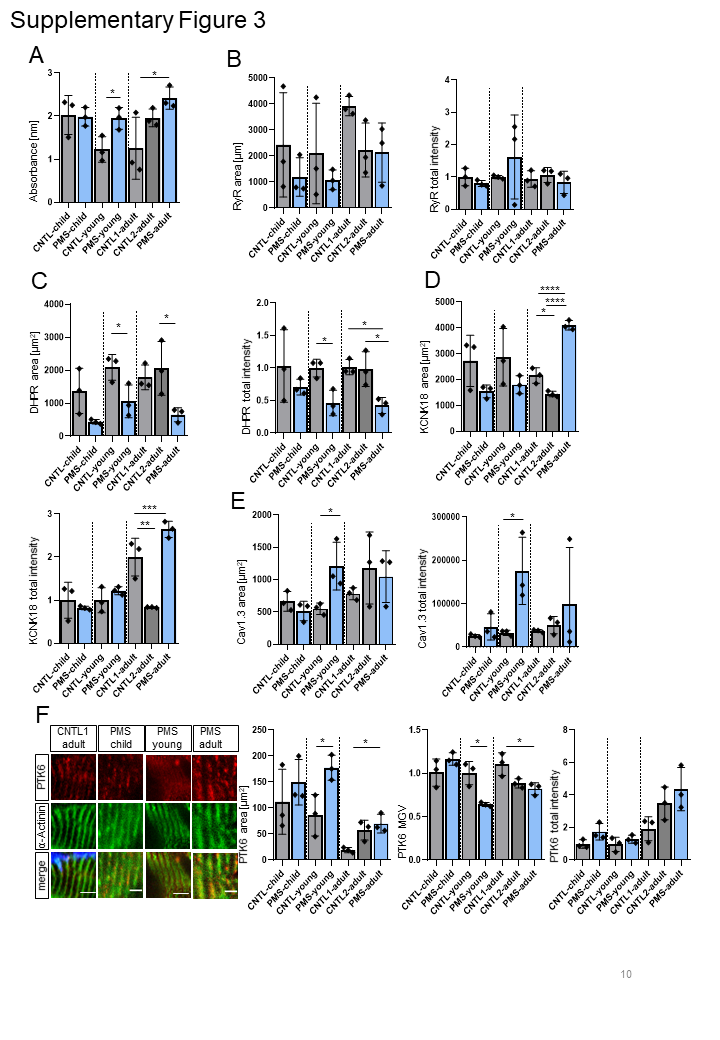

Supplement: Supplementary file 2 [file Image3.TIF]

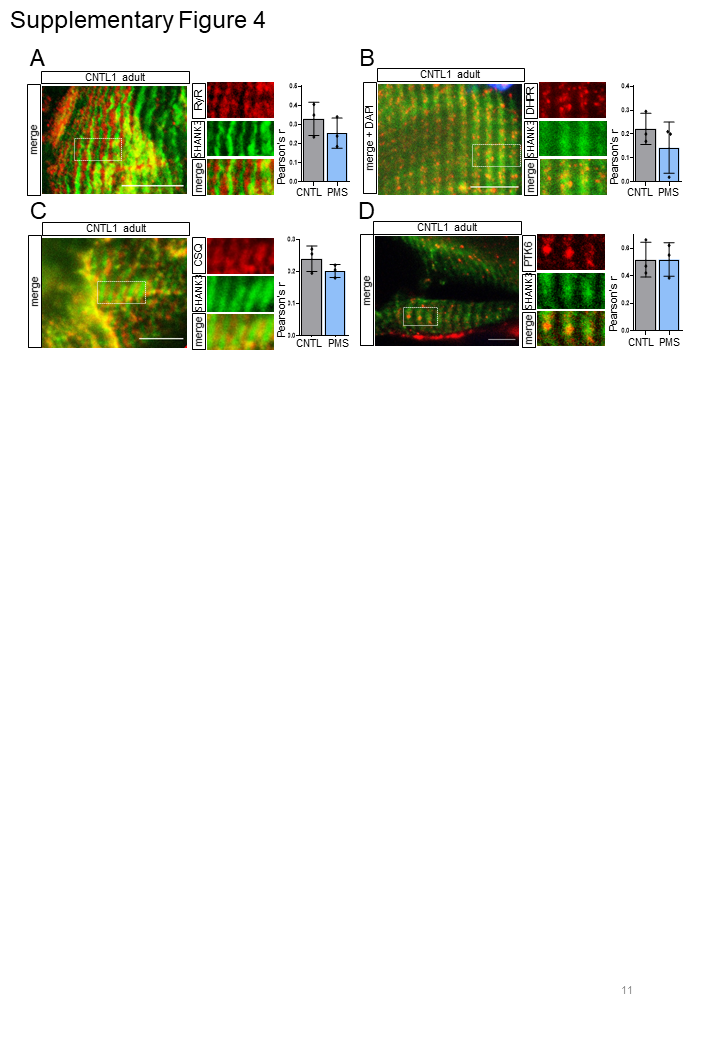

Supplement: Supplementary file 3 [file Image4.TIF]

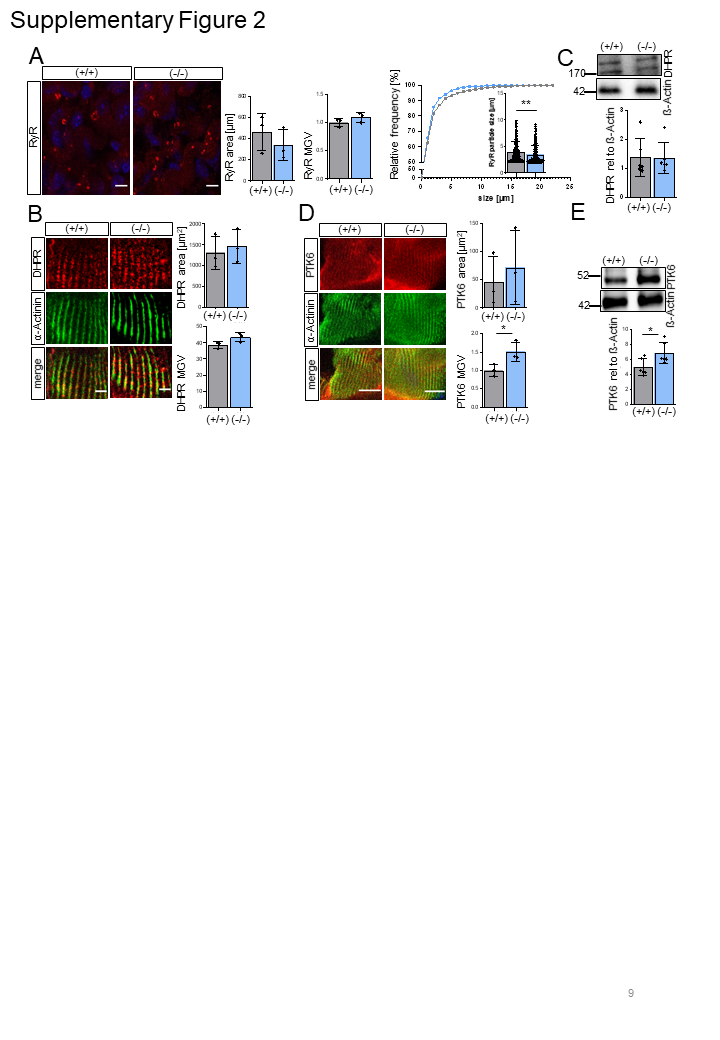

Supplement: Supplementary file 4 [file Image2.TIF]

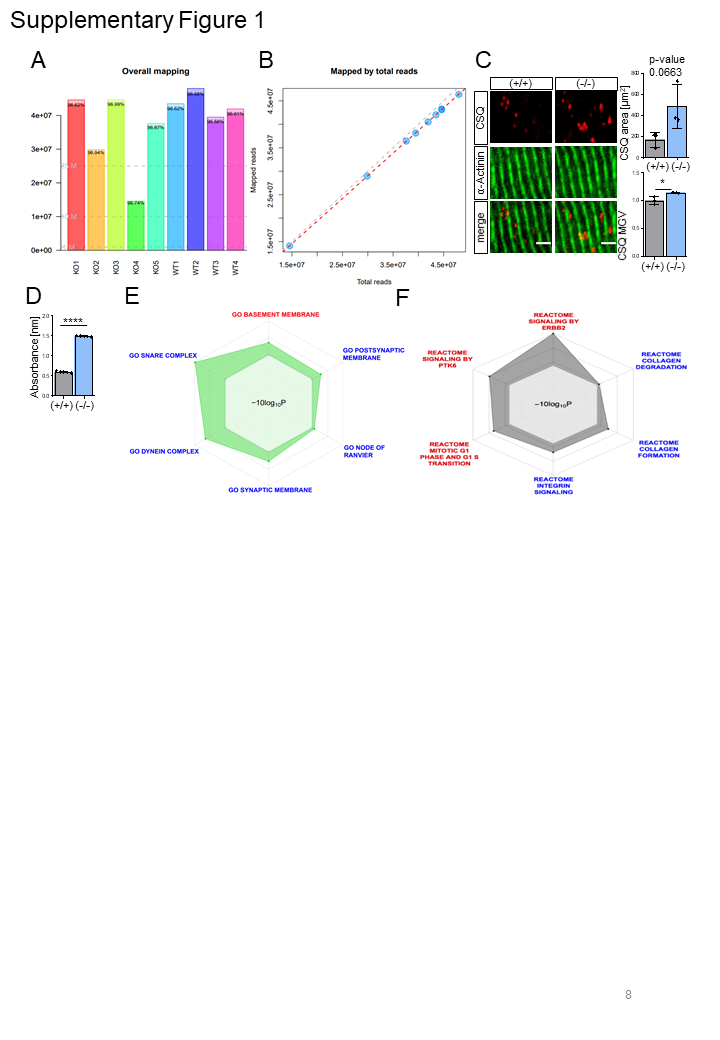

Supplement: Supplementary file 5 [file Image1.TIF]

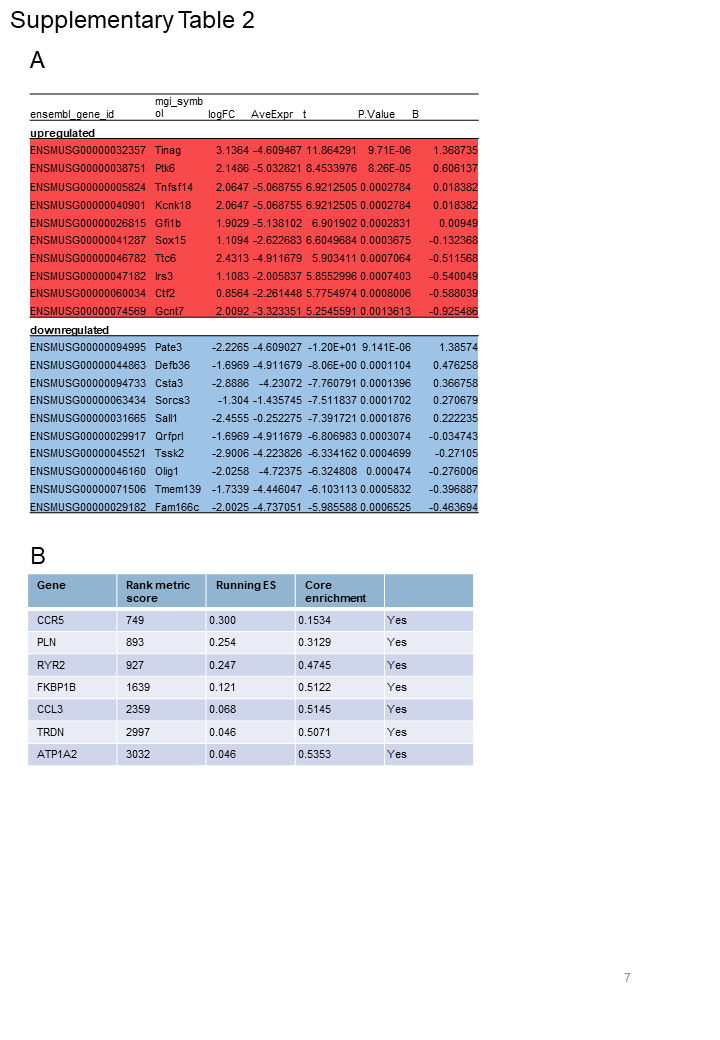

Supplement: Supplementary file 6 [file Image7.TIF]

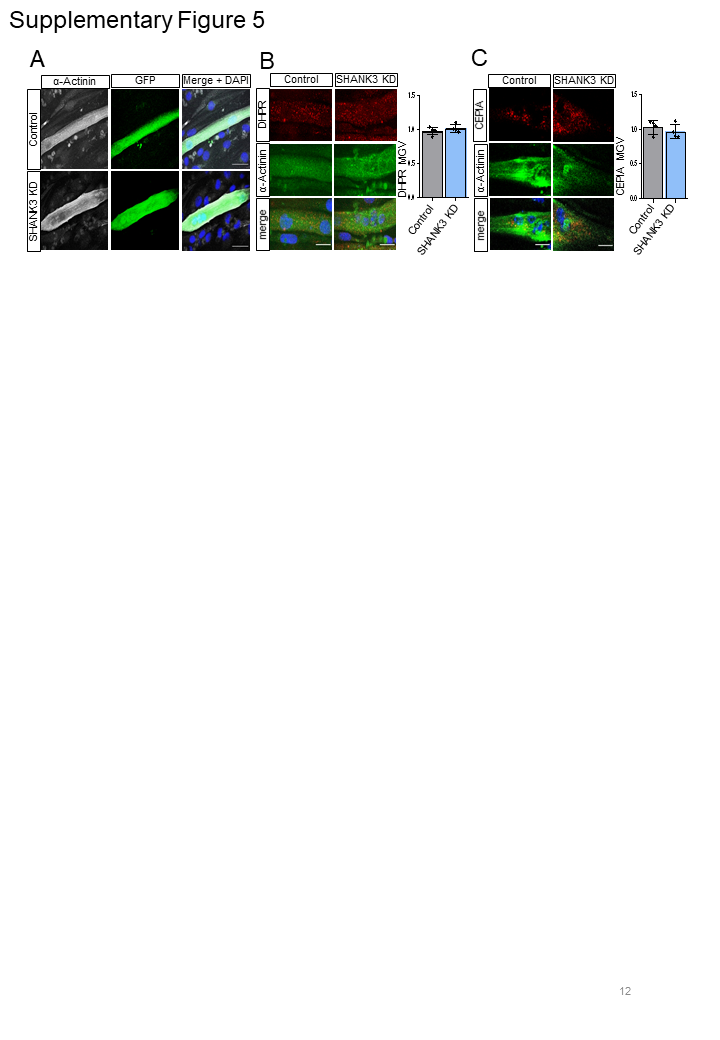

Supplement: Supplementary file 7 [file Image5.TIF]
