# Supplementary material for: Shank3 related muscular hypotonia is accompanied by increased intracellular calcium concentrations and ion channel dysregulation in striated muscle tissue
Source: Front Cell Dev Biol. 2023 Sep 6;11:1243299. doi: 10.3389/fcell.2023.1243299 (PMC10511643; doi:10.3389/fcell.2023.1243299)
Supplement: Supplementary file 8 [file DataSheet1.docx]

Supplementary Material

**Shank3 related muscular hypotonia is accompanied by increased intracellular Calcium concentrations and ion channel dysregulation in striated muscle tissue**

**Berra Yildiz^1,2#^, Lisa Schiedt^1^, Medhanie Mulaw^3^, Jürgen Bockmann^1^, Sarah Jesse^5^, Anne-Kathrin Lutz^1^, Tobias M. Boeckers^1,4*^**

^1^ Institute for Anatomy and Cell Biology, Ulm University, 89081 Ulm, Germany

^2^ International Graduate School in Molecular Medicine, IGradU, 89081, Ulm, Germany. ^3^ Institute of Molecular Medicine, Ulm University, 89081 Ulm, Germany. ^4^ DZNE, Ulm Site, 89081 Ulm, Germany ^5^ Neurologie, Universitätsklinikum Ulm, RKU, 89081 Ulm

*** Correspondence:**

Corresponding Author:
Dr. Tobias M. Boeckers
tobias.boeckers@uni-ulm.de

# Supplementary Figures and Tables

**Supplementary Table 1 non-protein coding DEGs of P0-1 *Shank3Δ11(−/−)* mice muscle RNA Sequencing.** Table demonstrate the top 20 up- and down-regulated DEGs for non-protein coding transcript variants. DEG: Differentially Expressed Gene.

**Supplementary Table 2 protein coding DEGs of P0-1 *Shank3****Δ****11(−/−)* mice muscle RNA Sequencing.** Table demonstrate the top 10 protein-coding genes. DEG: Differentially Expressed Gene.

**Supplementary Figure 1 Changes in calcium storage and gene expression in *Shank3****Δ****11(−/−)* mice. A)** Barplot displays the read count of each mouse's RNA-Sequencing results. **B)** Total number of mapped reads from KO2 and KO4 suggest lower library quality, hence those were excluded from the entire analysis. **C)** Immunofluorescence images of skeletal muscle tissue of P0-1 *Shank3Δ11(−/−)* and *Shank3(+/+)* mice for CSQ accumulation (red) and α-Actinin (green). *n*= 3 animals per genotype. Means ± SEM, Student’s Unpaired *t* test, area p=0.0663, MGV p=0.0257. Scale bar 5 µm. **D)** EDTA absorbance at 310 nm indicating total calcium concentration in the skeletal muscle. *n*= 5 animals per genotype. Means ± SEM, Student’s t test, EDTA p=0.0001. **e)** Principal component analyses of transcriptomic approach data from three Shank3Δ11(−/−) and four Shank3(+/+) animals. Each dot represents a biological replicate.  **E)** Up-and downregulated pathways of the GO cellular Component (CC). **F)** Radarplot representing selected up- or downregulated Reactome cluster. EDTA: Ethylenediaminetetraacetic acid, GO: Gene Ontology, KO: Knock-out, MGV: Mean Gray Value, SEM: Standard Error of the Mean.

**Supplementary Figure 2 Histological analysis show changed sarcolemmal receptor expression in skeletal muscles in *Shank3****Δ****11(−/−)* mice. A)** Immunofluorescence images of skeletal muscle tissue of P0-1 *Shank3Δ11(−/−)* and *Shank3(+/+)* mice for RyR (red) and α-Actinin (green). *n*= 3 animals per genotype. Means ± SEM, Student’s Unpaired *t* test, area p=0.4038, MGV p=0.2488, particle size p=0.0053. Scale bar 10 µm. **B)** Immunofluorescence images of skeletal muscle tissue of P0-1 *Shank3Δ11(−/−)* and *Shank3(+/+)* mice for DHPR (red) and α-Actinin (green). *n*= 3 animals per genotype. Means ± SEM, Student’s Unpaired *t* test, area p=0.6560, MGV p=0.0651 Scale bar 10 µm. **C)** Western blot analysis of DHPR and ß-Actin in skeletal muscle tissue lysates. *n*= 6 animals per genotype. Means ± SEM, Student’s Unpaired *t* test, p=0.9257. **D)** Immunofluorescence images of skeletal muscle tissue of P0-1 *Shank3Δ11(−/−)* and *Shank3(+/+)* mice for PTK6 (red) and α-Actinin (green). *n*= 3 animals per genotype. Means ± SEM, Student’s Unpaired *t* test, area p=0.6005, MGV p=0.0442. Scale bar 10 µm. **E)** Western blot analysis of PTK6 and ß-Actin in skeletal muscle tissue lysates. *n*= 6 animals per genotype. Means ± SEM, Student’s Unpaired *t* test, p=0.0268. DHPR: Dihydropyridine receptor, MGV: Mean Gray Value, PTK6: Phosphotyrosine kinase 6, RyR: Ryanodine receptor, SEM: Standard Error of the Mean.

**Supplementary Figure 3 Modified Sarcolemmal Receptor Expression in patients with PMS. A)** EDTA absorbance at 310 nm indicating total calcium concentration in the skeletal muscle. *n* equals number of technical replicates. Means ± SEM, Student’s *t* test for child and young and one-way ANOVA and Tukey’s multiple comparisons test for adult, child: p=0.8855, young: p=0.0329 and adult C1 vs. C2: p=0.2221, C1 vs. PMS: p=0.0469 and C2 vs. PMS: p=0.4791. **B)** Immunofluorescence analysis of skeletal muscle tissue of human biopsies for RyR area and total intensity. *n* equals number of technical replicates. Means ± SEM, Student’s Unpaired *t* test for child and young and one-way ANOVA and Tukey’s multiple comparisons test for adult, RyR area child: p=0.03744, young: p=0.4233 and adult C1 vs. C2: p=0.1370, C1 vs. PMS: p=0.1161 and C2 vs. PMS: p=0.9908, RyR total intensity: child: p=0.2989, young: p=0.4529 and adult C1 vs. C2: p=0.8591, C1 vs. PMS: p=0.8948 and C2 vs. PMS: p=0.6119. **C)** Immunofluorescence analysis of skeletal muscle tissue of human biopsies for DHPR area and total intensity. *n* equals number of technical replicates. Means ± SEM, Student’s Unpaired *t* test for child and young and one-way ANOVA and Tukey’s multiple comparisons test for adult, DHPR area child: p=0.0789, young: p=0.0480 and adult C1 vs. C2: p=0.8099, C1 vs. PMS: p= 0.0885 and C2 vs. PMS: p=0.0404, DHPR total intensity: child: p= 0.3806, young: p= 0.0177 and adult C1 vs. C2: p= 0,9796, C1 vs. PMS: p= 0,0176 and C2 vs. PMS: p= 0,0219. **D)** Immunofluorescence analysis of skeletal muscle tissue of human biopsies for KCNK18 area and total intensity. *n* equals number of technical replicates. Means ± SEM, Student’s Unpaired *t* test for child and young and one-way ANOVA and Tukey’s multiple comparisons test for adult, KCNK18 area child: p=0.1139, young: p=0.1892 and adult C1 vs. C2: p=0.0146, C1 vs. PMS: p<0.0001 and C2 vs. PMS: p<0.0001, KCNK18 total intensity: child: p=0.5020, young: p=0.2772 and adult C1 vs. C2: p=0.0049, C1 vs. PMS: p=0.0643 and C2 vs. PMS: p=0.0005. **E)** Immunofluorescence analysis of skeletal muscle tissue of human biopsies for Ca_V_1.3 area and total intensity. *n* equals number of technical replicates. Means ± SEM, Student’s Unpaired *t* test for child and young and one-way ANOVA and Tukey’s multiple comparisons test for adult, Ca_V_1.3 area child: p=0.2925, young: p=0.0399 and adult C1 vs. C2: p=0.4884, C1 vs. PMS: p=0.7137 and C2 vs. PMS: p=0.9134, Ca_V_1.3 total intensity: child: p=0.3112, young: p=0.0335 and adult C1 vs. C2: p=0.9748, C1 vs. PMS: p=0.6078 and C2 vs. PMS: p=0.7299. **F)** Immunofluorescence analysis of skeletal muscle tissue of human biopsies for PTK6 area, MGV and total intensity. *n* equals number of technical replicates. Means ± SEM, Student’s Unpaired *t* test for child and young and one-way ANOVA and Tukey’s multiple comparisons test for adult, PTK6 area child: p=0.4396, young: p=0.0277 and adult C1 vs. C2: p=0.0526, C1 vs. PMS: p=0.0162 and C2 vs. PMS: p=0.6037, KCNK18 MGV: child: p=0.1938, young: p=0.0102 and adult C1 vs. C2: p=0.0553, C1 vs. PMS: p=0.0208 and C2 vs. PMS: p=0.7038, KCNK18 total intensity: child: p=0.0828, young: p=0.3256 and adult C1 vs. C2: p=0.2456, C1 vs. PMS: p=0.0670 and C2 vs. PMS: p=0.5892. C: Control, CaV1.3: Voltage-gated calcium channel, DHPR: Dihydropyridine receptor, EDTA: Ethylenediaminetetraacetic acid, KCNK18: Calcium-gated potassium channel, PMS: PhelanMc-Dermid Syndrome, PTK6: Phosphotyrosine kinase 6, RyR: Ryanodine receptor, SEM: Standard Error of the Mean.

**Supplementary Figure 4 Human skeletal muscle tissue revealed no co-localization between SHANK3 and sarcolemmal receptors. A)** Expansion Microscopy of human skeletal muscle sections from control and PMS patients for RyR (red) and SHANK3 (green). *n* equals number of technical replicates. Means ± SEM, Student’s Unpaired *t* test, Pearson´s r: p=0.3293. Scale bar 10 µm. **B)** Expansion Microscopy of human skeletal muscle sections from control and PMS patients for DHPR (red) and SHANK3 (green). *n* equals number of technical replicates. Means ± SEM, Student’s Unpaired *t* test, Pearson´s r: p=0,3397. Scale bar 10 µm. **C)** Expansion Microscopy of human skeletal muscle sections from control and PMS patients for CSQ (red) and SHANK3 (green). *n* equals number of technical replicates. Means ± SEM, Student’s Unpaired *t* test, Pearson´s r: p=0.2103. Scale bar 10 µm. **D)** Expansion Microscopy of human skeletal muscle sections from control and PMS patients for PTK6 (red) and SHANK3 (green). *n* equals number of technical replicates. Means ± SEM, Student’s Unpaired *t* test, Pearson´s r: p=0.9918. Scale bar 10 µm. DHPR: Dihydropyridine receptor, MGV: Mean Gray Value, PMS: PhelanMc-Dermid Syndrome, PTK6: Phosphotyrosine kinase 6, RyR: Ryanodine receptor, SEM: Standard Error of the Mean.

**Supplementary Figure 5 Intensity of DHPR and CEPIA are unchanged in C2C12 Myotube Cells. A)** Immunofluorescence images of GFP (control) or SHANK3 siRNA transfected C2C12 myotubes for α-Actinin (gray) and GFP (green). Scale bar 100 μm. **B)** Immunofluorescence images of C2C12 myotube cell line for DHPR (red) and α-Actinin (green). *n* equals number of technical replicates. Means ± SEM, Student’s Unpaired *t* test DHPR MGV: p= 0.3714. Scale bar 50 µm. **C)** Immunofluorescence images of C2C12 myotube cell line for CEPIA (red) and α-Actinin (green). *n* equals number of technical replicates. Means ± SEM, Student’s Unpaired *t* test CEPIA MGV: p= 0.3953. Scale bar 50 µm. CEPIA: Calcium-measuring organelle-entrapped protein indicator, DHPR: Dihydropyridine receptor, MGV: Mean Gray Value, SEM: Standard Error of the Mean.
